# Crystal structure of 1-methane­sulfonyl-1,2,3,4-tetra­hydro­quinoline

**DOI:** 10.1107/S2056989014025353

**Published:** 2015-01-01

**Authors:** S. Jeyaseelan, S. L. Nagendra Babu, G. Venkateshappa, P. Raghavendra Kumar, B. S. Palakshamurthy

**Affiliations:** a Department of Physics, St Philomenas College (Autonomous), Mysore, Karnataka 570 015, India; b Department of Studies and Research in Physics, U.C.S., Tumkur University, Tumkur, Karnataka 572 103, India; c Department of Chemistry, Tumkur University, Tumkur, Karnataka 572 103, India

**Keywords:** crystal structure, 1,2,3,4-tetra­hydro­quinoline, physiological activities, photosensitizers

## Abstract

In the title compound, C_10_H_13_NO_2_S, the heterocyclic ring adopts a half-chair conformation and the bond-angle sum at the N atom is 347.9°. In the crystal, inversion dimers linked by pairs of C—H⋯O hydrogen bonds generate *R*_2_^2^(8) loops.

## Related literature   

For background to tetra­hydro­quinolines, see: Chulakov *et al.* (2012 ▸); Kadutskii *et al.* (2012 ▸); Katritsky *et al.* (1996 ▸); Keith *et al.* (2001 ▸). For a related structure, see: Jeyaseelan *et al.* (2014 ▸).
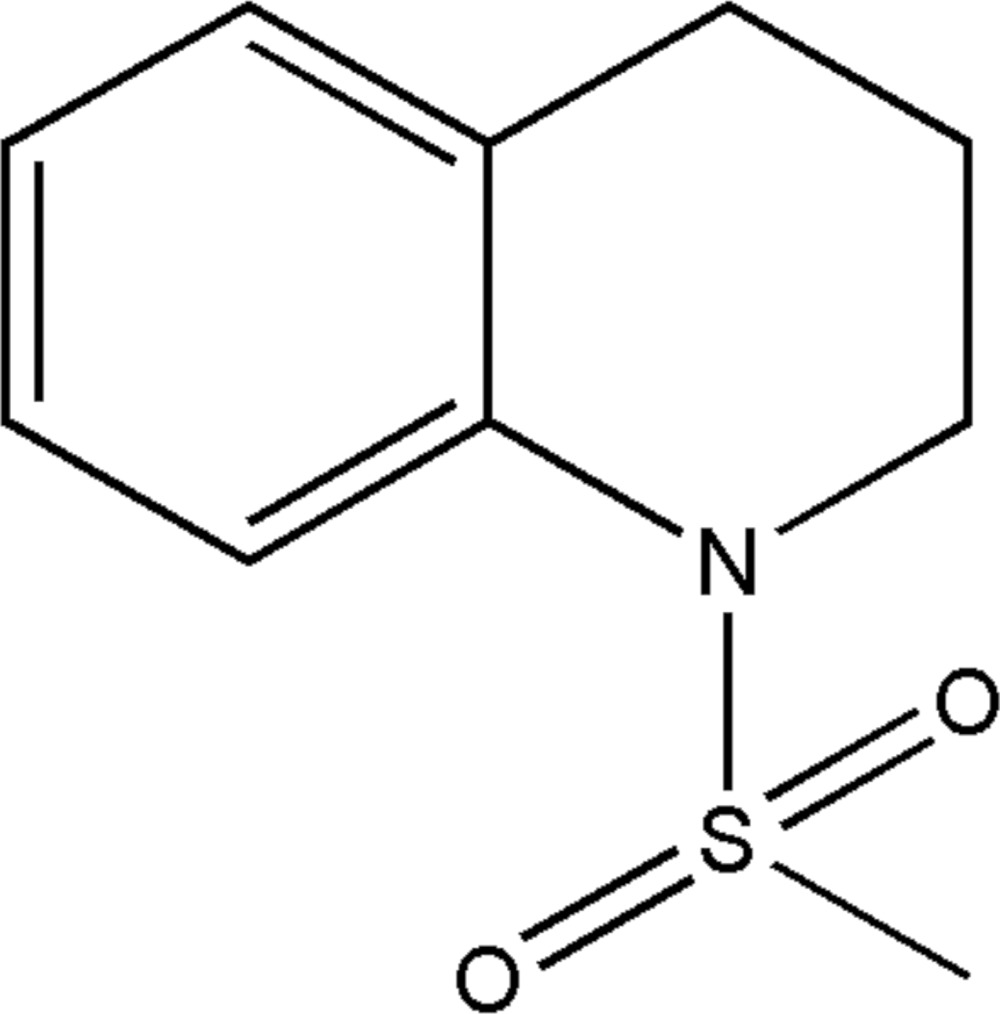


## Experimental   

### Crystal data  

C_10_H_13_NO_2_S
*M* *_r_* = 211.27
Triclinic, 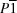

*a* = 5.5865 (2) Å
*b* = 9.2195 (4) Å
*c* = 10.1924 (4) Å
α = 85.798 (2)°
β = 84.686 (2)°
γ = 77.166 (2)°
*V* = 508.89 (4) Å^3^
*Z* = 2
Mo *K*α radiation
μ = 0.29 mm^−1^
*T* = 294 K
0.24 × 0.20 × 0.16 mm

### Data collection  

Bruker APEXII CCD diffractometer
Absorption correction: multi-scan (*SADABS*; Bruker, 2013 ▸) *T*_min_ = 0.933, *T*_max_ = 0.955
7417 measured reflections
1973 independent reflections
1844 reflections with *I* > 2σ(*I*)
*R*_int_ = 0.042

### Refinement  

*R*[*F* ^2^ > 2σ(*F* ^2^)] = 0.038
*wR*(*F* ^2^) = 0.106
*S* = 1.07
1973 reflections
128 parameters
H-atom parameters constrained
Δρ_max_ = 0.24 e Å^−3^
Δρ_min_ = −0.31 e Å^−3^

### 

Data collection: *APEX2* (Bruker, 2013 ▸); cell refinement: *SAINT* (Bruker, 2013 ▸); data reduction: *SAINT*; program(s) used to solve structure: *SHELXS97* (Sheldrick, 2008 ▸);; program(s) used to refine structure: *SHELXL2014* (Sheldrick, 2008 ▸); molecular graphics: *ORTEP-3 for Windows* (Farrugia, 2012 ▸) and *Mercury* (Macrae *et al.*, 2008 ▸); software used to prepare material for publication: *SHELXL2014*.

## Supplementary Material

Crystal structure: contains datablock(s) I. DOI: 10.1107/S2056989014025353/hb7314sup1.cif

Structure factors: contains datablock(s) I. DOI: 10.1107/S2056989014025353/hb7314Isup2.hkl

Supporting information file. DOI: 10.1107/S2056989014025353/hb7314Isup3.cml

**Fig. 1.**
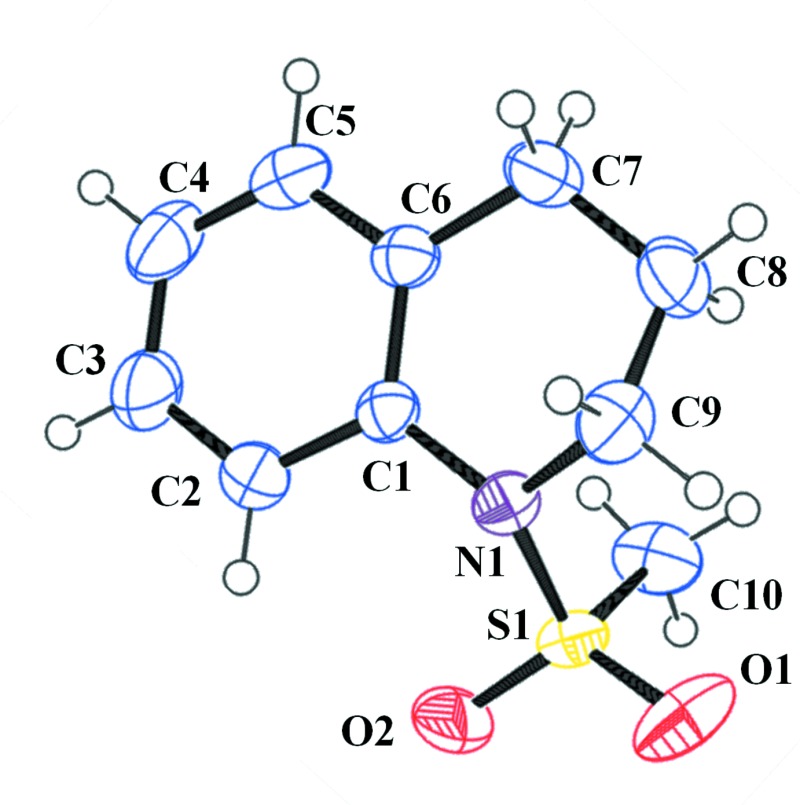
The molecular structure of the title compound, showing displacement ellipsoids drawn at the 50% probability level.

. DOI: 10.1107/S2056989014025353/hb7314fig1.tif
The mol­ecular structure of the title compound, showing displacement ellipsoids drawn at the 50% probability level.

**Fig. 2.**
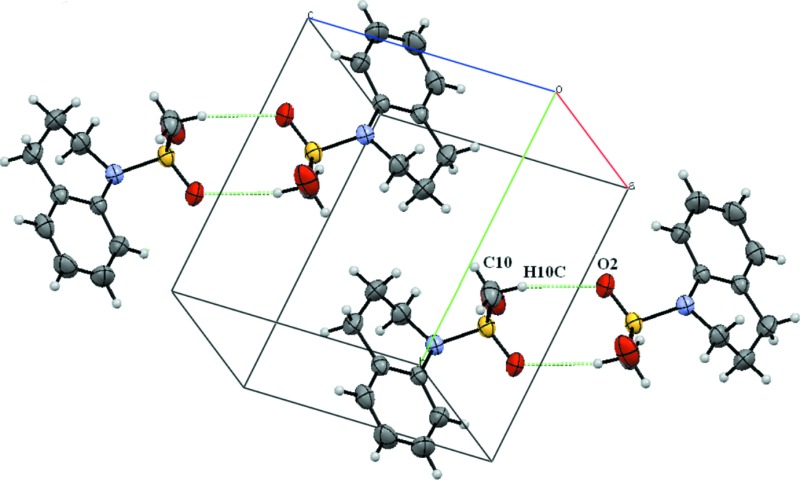
The molecular packing of the title compound, dashed lines indicates the inversion dimers linked by pairs of C—H···O hydrogen bonds with R22(8) ring motifs.

. DOI: 10.1107/S2056989014025353/hb7314fig2.tif
The mol­ecular packing of the title compound, dashed lines indicates the inversion dimers linked by pairs of C—H⋯O hydrogen bonds with 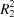
(8) ring motifs.

CCDC reference: 1034951

Additional supporting information:  crystallographic information; 3D view; checkCIF report

## Figures and Tables

**Table 1:** Hydrogen-bond geometry (, )

*D*H*A*	*D*H	H*A*	*D* *A*	*D*H*A*
C10H10*C*O2^i^	0.96	2.50	3.431(2)	164

Symmetry code: (i) 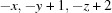.

## supplementary crystallographic information

### S1. Chemical context

Derivatives of tetra­hydro­quinolines display a wide range of physiological activities, they been found to be pesticides, anti­oxidants, photosensitizers, and dyes (Katritsky *et al.*, 1996). Heterocyclic compounds of 1,2,3,4-tetra­hydro­quinoline derivatives play important role in synthesize efficient kinetic resolution with predominant (S,S)-(R,R)-diastereoisomers (Chulakov *et al.*, 2012), optically active camphor moieties (Kadutskii *et al.*, 2012), and biologically active compounds, synthetic inter­mediates (Keith *et al.*, 2001).

In due course of our study, we have synthised a series of 1,2,3,4-tetra­hydro­quinoline with derivatives of suloponyl chlorides they exhibit a few pharmacological activities (our unpublished data). As a part of our study we have undertaken crystal structure determination of the title compound and the results are compared with crystal structure of 1-tosyl-1,2,3,4-tetra­hydro­quinoline­(II) (Jeyaseelan *et al.*, 2014) .

### S2. Structural commentary

The molecular structure of the title compound(I) is shown in Fig. 1. In both the compounds (I) and (II), the C1/C6–C9/N1 rings are in a half-chair conformation, with the methyl­ene C9 atom as the flap, but the bond-angle sum at the N atom in the compound (I) and (II) are 347.9° and 350.2°, respectively.

### S3. Supra­molecular features

In the crystal, inversion dimers linked by pairs of C10—H10C···O2 hydrogen bonds generate R_2_^2^(8) ring motifs.

### S4. Synthesis and crystallization

To a stirred solution of 1,2,3,4-tetra­hydro­quinoline (10 mmol) in 30 ml dry methyl­ene dichloride, tri­ethyl­amine (15 mmol) was added at 0 - 5°C. To this reaction mixture methane­sulfonyl chloride (12 mmol) in 10 ml dry di­chloro­methane was added drop wise. After 2h of stirring at 15 - 20°C, the reaction mixture was washed with 5% Na_2_CO_3_ and brine. The organic phase was dried over Na_2_SO_4_ and then it was concentrated on vacuum to yield titled compound as colourless solid. The crude product was recrystallized from a slovent mixture of ethyl acetate and hexane­(1:2) to yield colourless prisms of (I).

### S5. Refinement details

Crystal data, data collection and structure refinement details are summarized in Table 1. The H atoms were positioned with idealized geometry using a riding model with C—H = 0.93-0.99 Å. All H-atoms were refined with isotropic displacement parameters (set to 1.2-1.5 times of the U eq of the parent atom).

### Crystal data

**Table:**

C_10_H_13_NO_2_S	*F*(000) = 224
*M**_r_* = 211.27	Prism
Triclinic, *P*1	*D*_x_ = 1.379 Mg m^−^^3^
Hall symbol: -P 1	Melting point: 414 K
*a* = 5.5865 (2) Å	Mo *K*α radiation, λ = 0.71073 Å
*b* = 9.2195 (4) Å	Cell parameters from 1844 reflections
*c* = 10.1924 (4) Å	θ = 2.0–26.0°
α = 85.798 (2)°	µ = 0.29 mm^−^^1^
β = 84.686 (2)°	*T* = 294 K
γ = 77.166 (2)°	Prism, colourless
*V* = 508.89 (4) Å^3^	0.24 × 0.20 × 0.16 mm
*Z* = 2	

### Data collection

**Table:**

Bruker APEXII CCD diffractometer	1973 independent reflections
Radiation source: fine-focus sealed tube	1844 reflections with *I* > 2σ(*I*)
Graphite monochromator	*R*_int_ = 0.042
Detector resolution: 1.09 pixels mm^-1^	θ_max_ = 26.0°, θ_min_ = 2.0°
phi and ω scans	*h* = −6→6
Absorption correction: multi-scan (*SADABS*; Bruker, 2013)	*k* = −11→11
*T*_min_ = 0.933, *T*_max_ = 0.955	*l* = −12→12
7417 measured reflections	

### Refinement

**Table:**

Refinement on *F*^2^	Primary atom site location: difference Fourier map
Least-squares matrix: full	Secondary atom site location: difference Fourier map
*R*[*F*^2^ > 2σ(*F*^2^)] = 0.038	Hydrogen site location: inferred from neighbouring sites
*wR*(*F*^2^) = 0.106	H-atom parameters constrained
*S* = 1.07	*w* = 1/[σ^2^(*F*_o_^2^) + (0.0543*P*)^2^ + 0.1542*P*] where *P* = (*F*_o_^2^ + 2*F*_c_^2^)/3
1973 reflections	(Δ/σ)_max_ = 0.001
128 parameters	Δρ_max_ = 0.24 e Å^−^^3^
0 restraints	Δρ_min_ = −0.31 e Å^−^^3^
0 constraints	

### Special details

**Table:**

Geometry. All e.s.d.'s (except the e.s.d. in the dihedral angle between two l.s. planes) are estimated using the full covariance matrix. The cell e.s.d.'s are taken into account individually in the estimation of e.s.d.'s in distances, angles and torsion angles; correlations between e.s.d.'s in cell parameters are only used when they are defined by crystal symmetry. An approximate (isotropic) treatment of cell e.s.d.'s is used for estimating e.s.d.'s involving l.s. planes.

### Fractional atomic coordinates and isotropic or equivalent isotropic displacement parameters (Å^2^)

**Table:**

	*x*	*y*	*z*	*U*_iso_*/*U*_eq_	
O1	−0.0188 (3)	0.52244 (15)	0.71208 (15)	0.0682 (4)	
C1	0.3459 (3)	0.12140 (16)	0.73875 (15)	0.0323 (3)	
C2	0.2249 (4)	0.0388 (2)	0.83231 (18)	0.0478 (4)	
H2	0.0635	0.0778	0.8639	0.057*	
C3	0.3448 (4)	−0.1007 (2)	0.8780 (2)	0.0620 (6)	
H3	0.2647	−0.1549	0.9413	0.074*	
C4	0.5827 (4)	−0.1601 (2)	0.8300 (2)	0.0593 (5)	
H4	0.6643	−0.2534	0.8619	0.071*	
C5	0.6980 (3)	−0.0810 (2)	0.73512 (18)	0.0480 (4)	
H5	0.8574	−0.1227	0.7022	0.058*	
C6	0.5840 (3)	0.06025 (17)	0.68628 (15)	0.0359 (4)	
C7	0.7133 (3)	0.1364 (2)	0.57329 (19)	0.0489 (4)	
H7A	0.8788	0.1355	0.5955	0.059*	
H7B	0.7273	0.0797	0.4954	0.059*	
C8	0.5850 (4)	0.2949 (2)	0.54063 (19)	0.0537 (5)	
H8A	0.6378	0.3244	0.4512	0.064*	
H8B	0.6293	0.3604	0.6001	0.064*	
C9	0.3091 (4)	0.3101 (2)	0.55315 (16)	0.0461 (4)	
H9A	0.2296	0.4127	0.5318	0.055*	
H9B	0.2648	0.2483	0.4903	0.055*	
N1	0.2186 (2)	0.26547 (14)	0.68785 (12)	0.0343 (3)	
C10	0.3540 (4)	0.4495 (2)	0.8543 (2)	0.0548 (5)	
H10A	0.4427	0.4978	0.7856	0.082*	
H10B	0.4619	0.3619	0.8890	0.082*	
H10C	0.2931	0.5165	0.9235	0.082*	
O2	−0.0312 (3)	0.33906 (15)	0.89749 (14)	0.0592 (4)	
S1	0.10556 (7)	0.39877 (4)	0.78957 (4)	0.03662 (17)	

### Atomic displacement parameters (Å^2^)

**Table:**

	*U*^11^	*U*^22^	*U*^33^	*U*^12^	*U*^13^	*U*^23^
O1	0.0777 (10)	0.0463 (8)	0.0683 (9)	0.0213 (7)	−0.0239 (8)	−0.0061 (7)
C1	0.0337 (7)	0.0307 (7)	0.0331 (7)	−0.0068 (6)	−0.0033 (6)	−0.0056 (6)
C2	0.0479 (10)	0.0421 (9)	0.0499 (10)	−0.0084 (8)	0.0107 (8)	−0.0023 (7)
C3	0.0783 (15)	0.0417 (10)	0.0591 (12)	−0.0096 (10)	0.0141 (10)	0.0071 (9)
C4	0.0768 (14)	0.0358 (9)	0.0578 (11)	0.0021 (9)	−0.0054 (10)	0.0022 (8)
C5	0.0419 (9)	0.0425 (9)	0.0553 (10)	0.0027 (7)	−0.0041 (8)	−0.0117 (8)
C6	0.0336 (8)	0.0364 (8)	0.0390 (8)	−0.0080 (6)	−0.0024 (6)	−0.0093 (6)
C7	0.0381 (9)	0.0537 (10)	0.0543 (10)	−0.0125 (8)	0.0100 (8)	−0.0084 (8)
C8	0.0626 (12)	0.0528 (11)	0.0455 (10)	−0.0200 (9)	0.0140 (9)	−0.0013 (8)
C9	0.0606 (11)	0.0445 (9)	0.0308 (8)	−0.0067 (8)	−0.0049 (7)	0.0003 (7)
N1	0.0350 (7)	0.0333 (7)	0.0336 (7)	−0.0043 (5)	−0.0030 (5)	−0.0042 (5)
C10	0.0488 (10)	0.0674 (12)	0.0529 (11)	−0.0158 (9)	−0.0023 (8)	−0.0261 (9)
O2	0.0526 (8)	0.0569 (8)	0.0645 (9)	−0.0122 (6)	0.0261 (7)	−0.0179 (7)
S1	0.0304 (2)	0.0346 (3)	0.0418 (3)	0.00108 (16)	−0.00305 (16)	−0.00688 (17)

### Geometric parameters (Å, º)

**Table:**

O1—S1	1.4227 (13)	C7—H7B	0.9700
C1—C2	1.396 (2)	C8—C9	1.511 (3)
C1—C6	1.398 (2)	C8—H8A	0.9700
C1—N1	1.4446 (18)	C8—H8B	0.9700
C2—C3	1.381 (3)	C9—N1	1.480 (2)
C2—H2	0.9300	C9—H9A	0.9700
C3—C4	1.379 (3)	C9—H9B	0.9700
C3—H3	0.9300	N1—S1	1.6446 (13)
C4—C5	1.369 (3)	C10—S1	1.7555 (18)
C4—H4	0.9300	C10—H10A	0.9600
C5—C6	1.394 (2)	C10—H10B	0.9600
C5—H5	0.9300	C10—H10C	0.9600
C6—C7	1.515 (2)	O2—S1	1.4279 (13)
C7—C8	1.505 (3)	S1—O1	1.4227 (13)
C7—H7A	0.9700		
			
C2—C1—C6	120.12 (15)	C9—C8—H8A	109.6
C2—C1—N1	120.16 (14)	C7—C8—H8B	109.6
C6—C1—N1	119.53 (13)	C9—C8—H8B	109.6
C3—C2—C1	120.02 (17)	H8A—C8—H8B	108.1
C3—C2—H2	120.0	N1—C9—C8	111.80 (14)
C1—C2—H2	120.0	N1—C9—H9A	109.3
C4—C3—C2	120.28 (18)	C8—C9—H9A	109.3
C4—C3—H3	119.9	N1—C9—H9B	109.3
C2—C3—H3	119.9	C8—C9—H9B	109.3
C5—C4—C3	119.56 (18)	H9A—C9—H9B	107.9
C5—C4—H4	120.2	C1—N1—C9	114.89 (12)
C3—C4—H4	120.2	C1—N1—S1	119.76 (10)
C4—C5—C6	122.06 (16)	C9—N1—S1	117.41 (10)
C4—C5—H5	119.0	S1—C10—H10A	109.5
C6—C5—H5	119.0	S1—C10—H10B	109.5
C5—C6—C1	117.87 (15)	H10A—C10—H10B	109.5
C5—C6—C7	119.39 (15)	S1—C10—H10C	109.5
C1—C6—C7	122.61 (15)	H10A—C10—H10C	109.5
C8—C7—C6	114.00 (14)	H10B—C10—H10C	109.5
C8—C7—H7A	108.8	O1—S1—O2	118.38 (10)
C6—C7—H7A	108.8	O1—S1—N1	106.54 (8)
C8—C7—H7B	108.8	O2—S1—N1	108.22 (7)
C6—C7—H7B	108.8	O1—S1—C10	108.39 (10)
H7A—C7—H7B	107.6	O2—S1—C10	107.06 (9)
C7—C8—C9	110.45 (15)	N1—S1—C10	107.85 (8)
C7—C8—H8A	109.6		
			
C6—C1—C2—C3	3.1 (3)	C6—C1—N1—C9	22.44 (19)
N1—C1—C2—C3	178.13 (17)	C2—C1—N1—S1	59.22 (18)
C1—C2—C3—C4	−1.0 (3)	C6—C1—N1—S1	−125.77 (13)
C2—C3—C4—C5	−1.1 (3)	C8—C9—N1—C1	−51.15 (19)
C3—C4—C5—C6	1.2 (3)	C8—C9—N1—S1	97.83 (15)
C4—C5—C6—C1	0.9 (3)	C1—N1—S1—O1	−176.90 (12)
C4—C5—C6—C7	−174.89 (18)	C9—N1—S1—O1	35.68 (15)
C2—C1—C6—C5	−3.1 (2)	C1—N1—S1—O1	−176.90 (12)
N1—C1—C6—C5	−178.07 (13)	C9—N1—S1—O1	35.68 (15)
C2—C1—C6—C7	172.62 (15)	C1—N1—S1—O2	−48.59 (13)
N1—C1—C6—C7	−2.4 (2)	C9—N1—S1—O2	163.98 (12)
C5—C6—C7—C8	−173.18 (16)	C1—N1—S1—O2	−48.59 (13)
C1—C6—C7—C8	11.2 (2)	C9—N1—S1—O2	163.98 (12)
C6—C7—C8—C9	−38.3 (2)	C1—N1—S1—C10	66.91 (14)
C7—C8—C9—N1	58.9 (2)	C9—N1—S1—C10	−80.52 (14)
C2—C1—N1—C9	−152.58 (15)		

### Hydrogen-bond geometry (Å, º)

**Table:**

*D*—H···*A*	*D*—H	H···*A*	*D*···*A*	*D*—H···*A*
C10—H10*C*···O2^i^	0.96	2.50	3.431 (2)	164

Symmetry code: (i) −*x*, −*y*+1, −*z*+2.
