# Supplementary figures and images for: Crystal structure of 1-methane­sulfonyl-1,2,3,4-tetra­hydro­quinoline
Source: Acta Crystallogr E Crystallogr Commun. 2015 Jan 1;71(Pt 1):o20. doi: 10.1107/S2056989014025353 (PMC4331909; doi:10.1107/S2056989014025353)

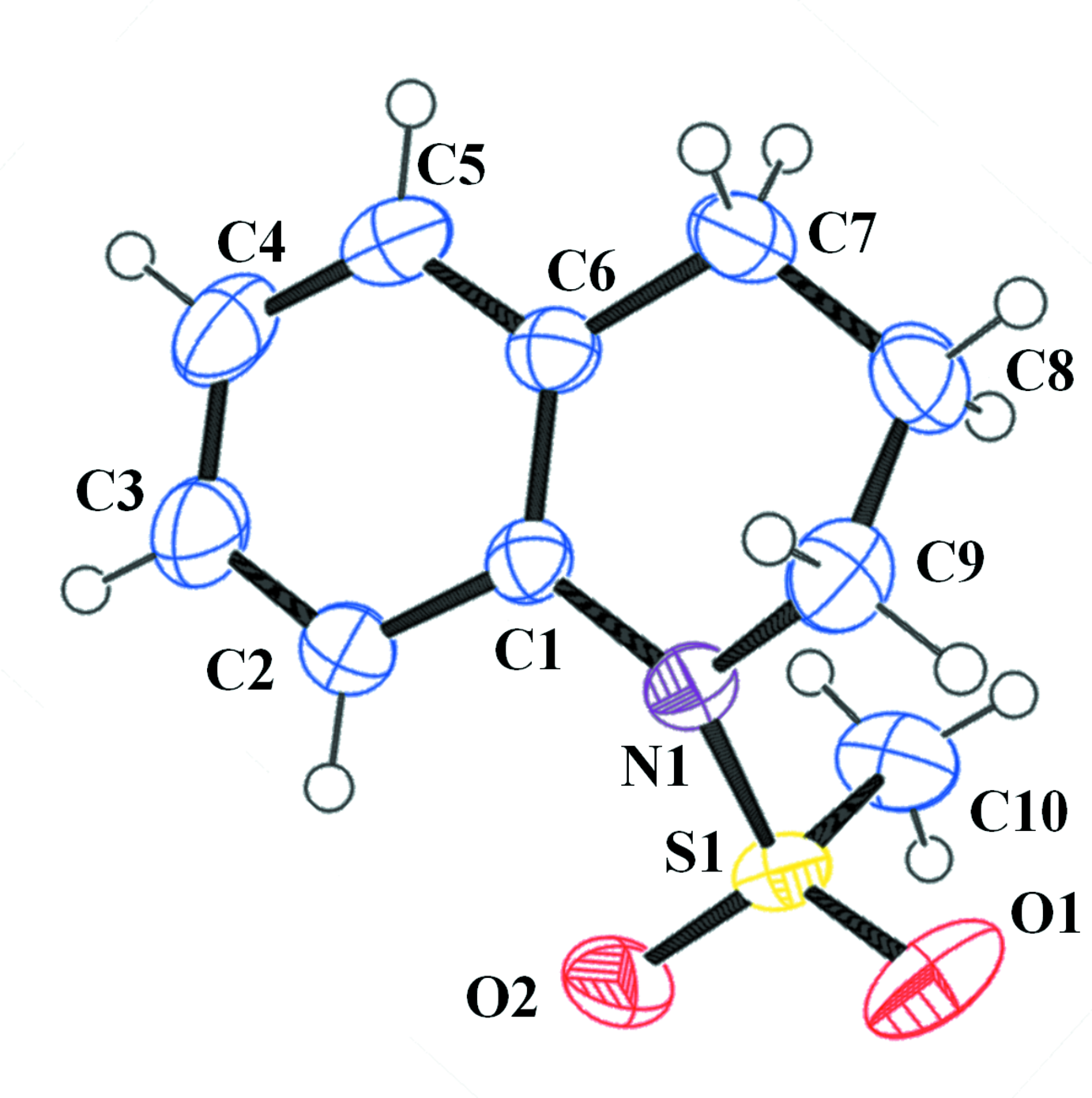

Supplement: Supplementary file 4 [file e-71-00o20-fig1.tif]

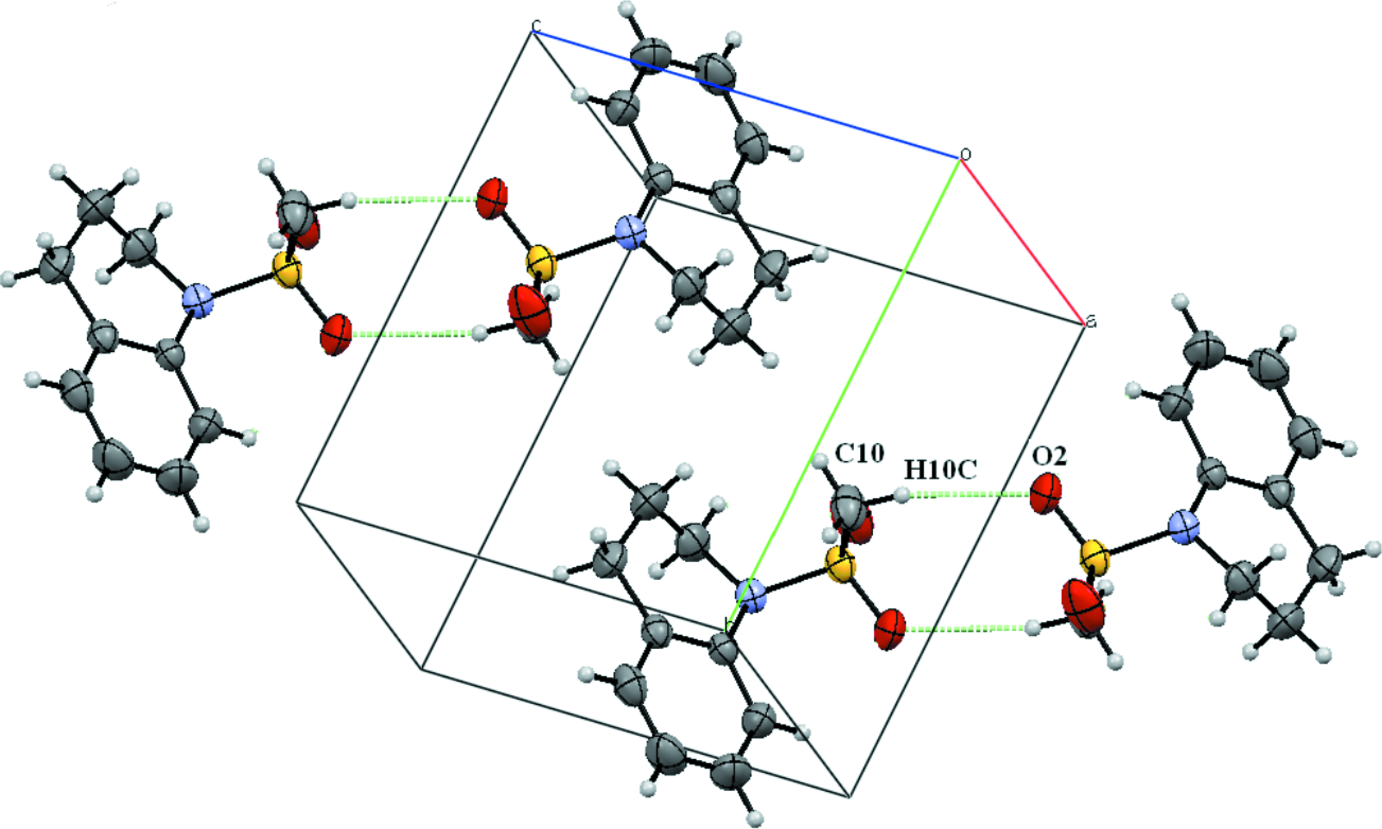

Supplement: Supplementary file 5 [file e-71-00o20-fig2.tif]
